# Examination of Imprinting Process with Molsidomine as a Template

**DOI:** 10.3390/molecules14062212

**Published:** 2009-06-17

**Authors:** Piotr Luliński, Dorota Maciejewska

**Affiliations:** Department of Organic Chemistry, Faculty of Pharmacy, Medical University of Warsaw, Banacha 1 Str., 02-097 Warsaw, Poland; E-mail: dorota.maciejewska@wum.edu.pl (D.M.)

**Keywords:** molsidomine, molecularly imprinted polymers, sustained release

## Abstract

Eight different functional monomers were used with ethylene glycol dimethacrylate as a cross-linker and molsidomine as a template to obtain molecularly imprinted polymers (MIPs). Non-covalent interactions between molsidomine and each functional monomer in DMSO prior to thermal bulk polymerization were utilized. On the basis of calculated imprinting factors, MIP prepared with *N*,*N’*-diallyltartaramide was chosen for further investigations. Examination of interactions in the prepolymerization complex between molsidomine and *N*,*N’*-diallyltartaramide was performed using the Job method. The absorbance of isomolar solutions reaching a maximum for the molar ratio of template to monomer equal to 1:4. Scatchard analysis was used for estimation of the dissociation constants and the maximum amounts of binding sites. The polymer based on *N*,*N’*-diallyltartaramide has two classes of heterogeneous binding sites characterized by two values of K_d_ and two B_max_: K_d_(1) = 1.17 mM^-1^ and B_max_(1) = 0.8 μmol/mg for the higher affinity binding sites, and K_d_(2) = 200 μM^-1^ and B_max_(2) = 2.05 μmol/mg for the lower affinity binding sites. Furthermore, effects of pH and organic solvent on binding properties of MIP and NIP were investigated, together with release of molsidomine from both MIP and NIP.

## Introduction

Molecular imprinting is a technique for preparing polymers of desired and predetermined selectivity [1]. Functional groups of template molecule (for which selectivity is desired) are involved in the interactions with monomer(s) prior to the polymerization process. Template molecules are removed after polymerization, leaving well-defined, three-dimensional cavities with spatially oriented functionalities, complementary to the target molecule, in the highly cross-linked polymer network. In their most common form, MIPs are prepared as a monolith which is then ground and sieved to the appropriate size of particles. Molecularly imprinted polymers can be applied in many areas such as chromatography or solid phase extraction (SPE) [2], as antibody mimics [3] or in catalysis [4].

In the last few years MIPs have been reported to be suitable for drug delivery systems, especially for controlled release devices of drugs with a narrow therapeutic index [5,6,7]. A polymeric network which is imprinted for a particular drug could improve drug delivery profiles by providing extended release times or increasing drug loading within the matrix. Extended release is mainly achieved via interactions of the drug with complementary functional groups organized around the imprinting site in the polymer. Selectivity of imprinted materials could be used to deliver a specific enantiomer of the drug. The combination of both the recognition and release properties of MIPs, could result in their usefulness in drug delivery systems. It is well-known that after appropriate post-synthetic preparation most imprinted polymers can be used for drug delivery because they are non-toxic and biocompatible [8,9].

**Figure 1:**
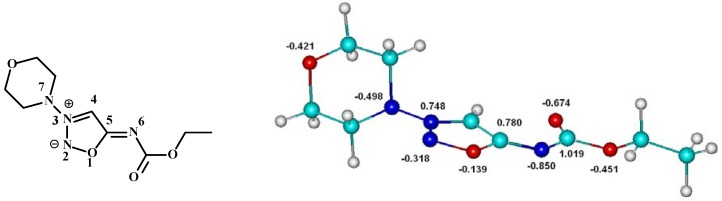
Chemical formula of molsidomine with formal charges in sydnone ring and optimized structure with ESP atomic charges.

Molsidomine [(*N*-ethoxycarbonyl)-3-(4-morpholino)sydnonimine, Figure 1], is an antiangial prodrug, an alternative to the organic nitrates widely used in the treatment of ischaemia coronary artery disease [10]. Molsidomine is metabolized in the liver to its active metabolite, linsidomine, next transformed into other intermediates, and finally forming a pharmacologically active nitric oxide. Nitric oxide should be released slowly to avoid toxicity [11].

We have applied DFT computation method with B3LYP/6-311G+(d,p) hybrid functional to get the ESP atomic charges (Brenneman model) for molsidomine [12]. The geometry of molsidomine was optimized about the expected configuration obtained by X-ray diffraction measurement [13].

The charge distribution at molsidomine atoms showed large charge separation as was expected. The N3 nitrogen atom and the C5 carbon atom in the sydnone ring are the electron-deficient atoms, and induce a strong polarization of the accompanying bonds. As a consequence at the nitrogen atoms N2, N6 and N7 the negative charges are localized (Figure 1). Such a structure containing a dipolar five membered heterocyclic ring with delocalized both the negative and the positive charges is called mesoionic [14]. The mesoionic structure of the template molecule could prevent the formation of selective binding sites in polymer matrix. We could suppose that the combination of a lot of complementary interactions take place between molsidomine and monomer: for example Coulomb and hydrogen bond types of interactions with participation of N and O atoms.

The main aim of this paper was the analysis of molsidomine imprinted polymers. The interactions between template and eight functional monomers were also examined. To the best of our knowledge, this is the first report on the interactions in the prepolymerization complex and imprinting process with molsidomine as a template. It is also the first attempt at the imprinting process using the sydnone mesoionic compound.

## Results and Discussion

### Choice of functional monomer

Eight kinds of imprinted polymers **MIP1** – **MIP8** and corresponding non-imprinted ones (**NIP1** – **NIP8**) were prepared using eight different monomers: methacrylic acid (**1**) and itaconic acid (**2**) with acidic groups, 2-hydroxyethyl methacrylate (**3**) and ethyl acrylate (**4**) with ester groups, acrylamide (**5**), methacrylamide (**6**), *N*,*N’*-methylenebisacrylamide (**7**) and *N*,*N’*-diallyltartaramide (**8**) with amide functionalities. 

**Scheme 1:**
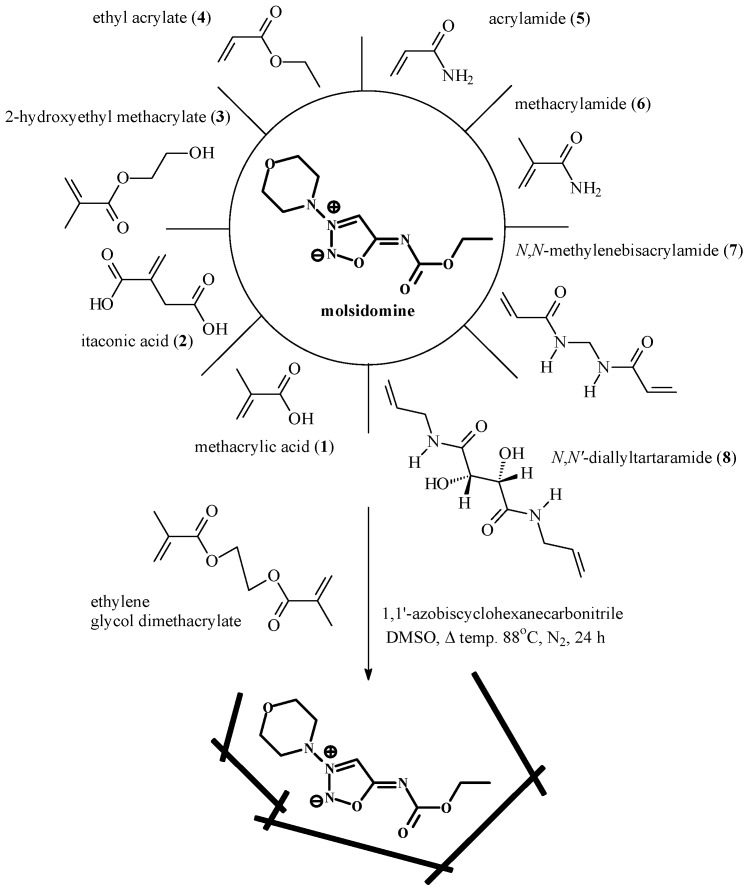
Reagents and conditions of MIP synthesis.

The template molecule, subsequent functional monomers, the cross-linker and initiator were dissolved in DMSO (Scheme 1) in a thin-walled glass tube. DMSO was chosen as a porogen due to the low solubility of molsidomine and functional monomers in many organic solvents. Molsidomine is soluble in DMSO and chloroform and practically insoluble in other organic solvents, whereas some monomers are insoluble in chloroform. The molar ratio of template to functional monomer and cross-linker was 1:4:16. The obtained solution was purged with nitrogen, the glass tube was sealed and incubated at 88 ^o^C for 24 h. Non-imprinted polymers were prepared in the same way, except the addition of template.

**Figure 2:**
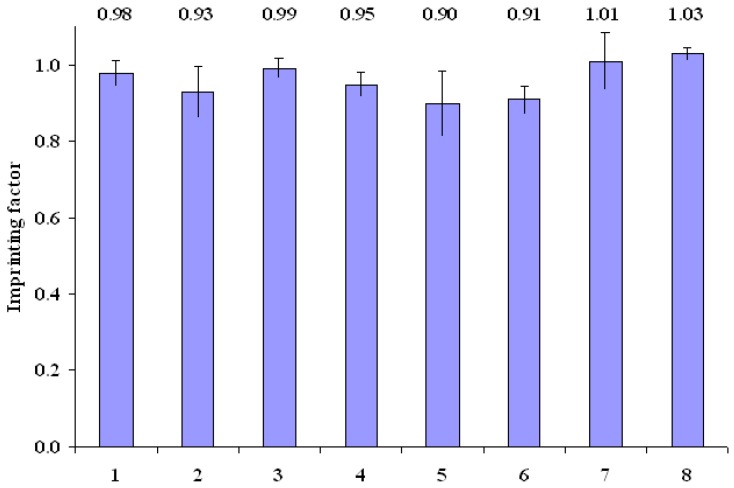
Imprinting factors of evaluated pairs of polymers in aqueous media.

The binding experiments were performed to estimate the quality of the obtained polymers. Stationary and dynamic binding experiments (see Experimental, subsections a and d) were carried out in aqueous media with molsidomine standard solutions of 0.7 and 0.07 μmol/mL. Imprinting factors calculated as the ratio of molsidomine amount bound to the imprinted polymer to molsidomine amount bound to control polymer, are shown in Figure 2. The values of the imprinting factors for all polymers are similar and are close to unity. In this experiment the specific binding sites were not revealed.

**Figure 3:**
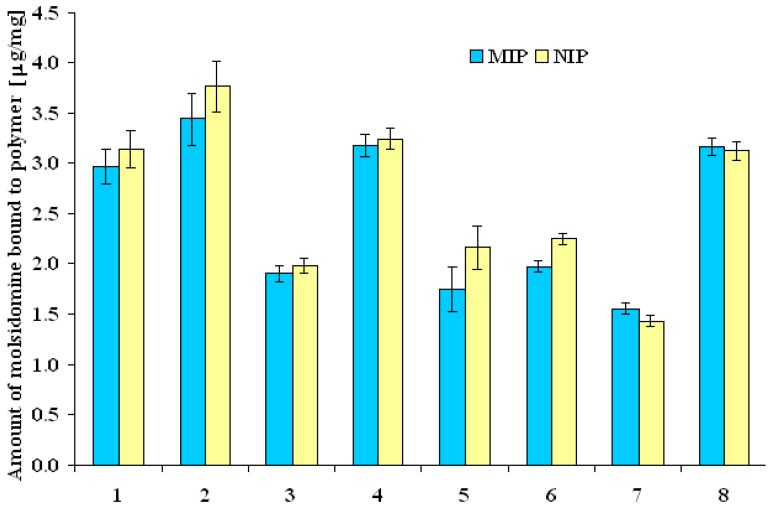
The amount of molsidomine bound to polymers in aqueous media.

Next, the capacity of the matrices was evaluated on the basis of dynamic binding experiments in aqueous media with molsidomine standard solution of 0.07 μmol/mL. The results are presented in Figure 3. It was demonstrated that a key role as concerns the capacity was played by the functional monomer. As it may be noted, the highest value of adsorption was observed for polymer prepared with itaconic acid (**MIP2**: 3.44 μg/mg and **NIP2**: 3.76 μg/mg), and the lowest value for polymer prepared with *N*,*N’*-methylenebisacrylamide (**MIP7**: 1.56 μg/mg and **NIP7**: 1.43 μg/mg). The small difference between the quantity of molsidomine bound to MIPs and NIPs was the evidence of high non-specific binding in water. Only for polymer **MIP8**, obtained from *N*,*N’*-diallyltartaramide (**8**), the capacity was sufficiently high and the imprinting factor was slightly higher than 1, and therefore **MIP8** was selected for further examination.

### Composition of the prepolymerization complex

**Figure 4:**
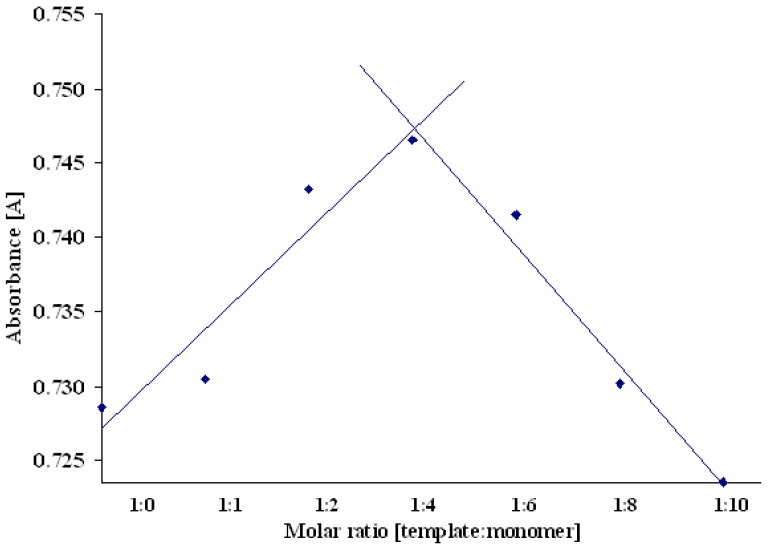
Job’s plot presenting the relations between absorbance and molar ratio of template to functional monomer.

It has been demonstrated that the imprinting procedure depends on the stability of the molecular assembles in the prepolymerization mixture. Therefore the analysis of molecular ratio in the possible complexes could be helpful in planning the polymer synthesis. The prepolymerization complex of molsidomine with *N*,*N’*-diallyltartaramide was examined by Job’s method. This well-known method is often used to determined the stoichiometry of many kind of complexes, and is applied with various analytical techniques i.e. ^1^H-NMR or UV-VIS spectroscopies. Job plot based on the spectral changes observed either for the host or the guest molecule [15,16,17]. We measured the absorbance of isomolar solutions of both components in methanol at wavelength λ_max_ = 317 nm. The choice of methanol (instead of DMSO used as a porogen) was dictated by the high absorbance of DMSO in the range of wavelength relevant for molsidomine. The resulting Job’s plot is presented in Figure 4.

The data obtained clearly show that the absorbance increases with the increase of molar ratio of *N*,*N’*-diallyltartaramide, reaching a maximum for the molar ratio of template to monomer equal to 1:4. The absorbance decreases at the higher molar fraction of monomer. This effect could be explained by some self-organization of monomer molecules at their higher molar concentrations. The most stable prepolymerization complex consists of one molecule of molsidomine and four molecules of *N*,*N’*-diallyltartaramide.

### MIP and NIP binding properties

In the binding properties many important factors are crucial. We have investigated three of them: the concentration of aqueous molsidomine standard solution, pH of solution, and solvent used for loading of molsidomine. Stationary and dynamic binding experiments were employed (see Experimental, subsections b and d).

**Figure 5:**
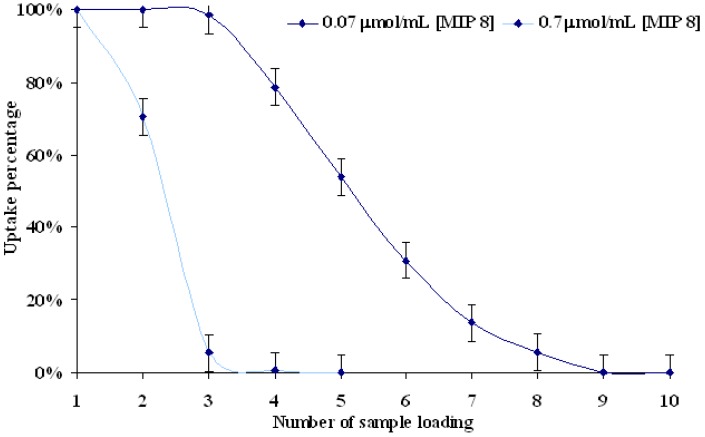
Study of complete saturation of **MIP8** at different concentrations of aqueous molsidomine standard solutions.

The first experiment was performed for two different concentrations of aqueous standard solutions: 0.7 and 0.07 μmol/mL in dynamic procedure. Successive portions (1 mL) of both standard solutions were put on matrices. The saturation of polymer matrix by molsidomine was considerably different in both 0.7 and 0.07 μmol/mL solutions. Complete saturations came after 9 and 4 portions for 0.07 and 0.7 μmol/mL solutions, respectively. Total amounts of molsidomine adsorbed from 0.7 μmol/mL solution were 11.79 μg/mg for **MIP8** and 11.57 μg/mg for **NIP8**, and from 0.07 μmol/mL solution were 3.17 μg/mg for **MIP8** and 3.12 μg/mg for **NIP8**. The percentage of molsidomine bound by **MIP8** and **NIP8** is reported in Figure 5.

**Figure 6:**
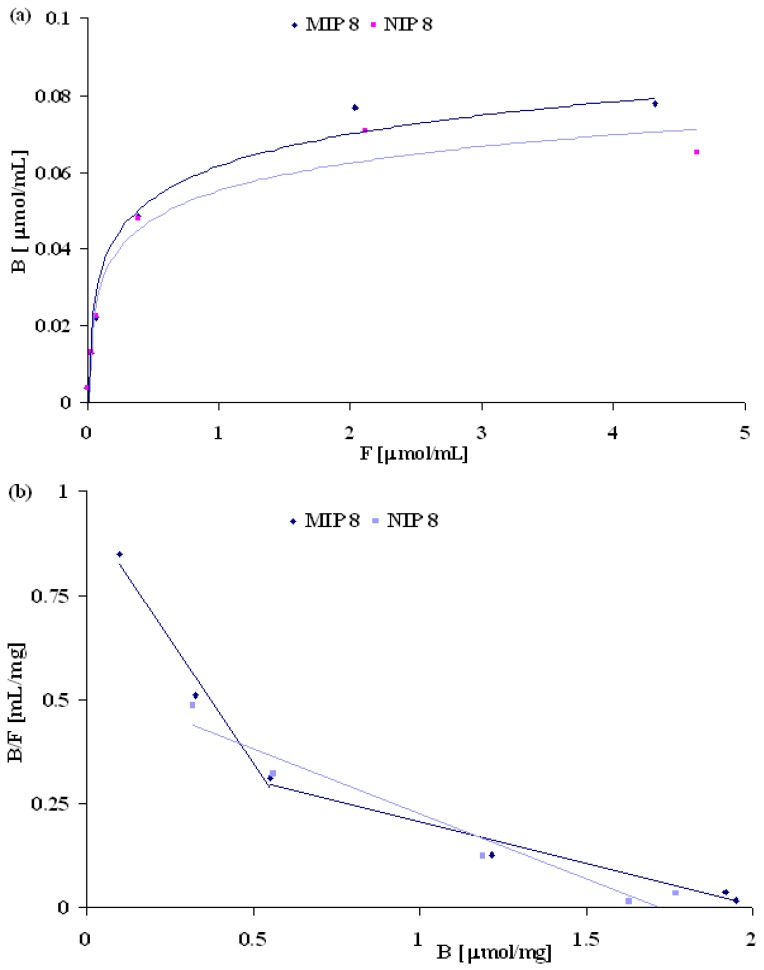
Binding isotherms and Scatchard plots obtained for **MIP8** and **NIP8**.

The binding characteristic of **MIP8** and **NIP8** was also estimated by Scatchard plots represented by the equation B/F = (B_max_ – B)/K_d_, where K_d_ is the dissociation constant and B_max_ is the maximum amount of binding sites. The concentrations of free (F) and bound (B) molsidomine were determined by measuring the unbound fraction in the solution [18,19]. The binding isotherms were determined in water solutions by adding a fixed amount of polymer to the various concentrations of molsidomine (0.01 to 6 μmol/mL) (Figure 6a). Only a slight difference in binding properties between **MIP8** and **NIP8** was noted. However, Scatchard plots presented in Figure 6b revealed two straight lines for **MIP8**, and only one such line for **NIP8**. This result is typical of imprinted polymers obtained by the non-covalent approach. Polymer **MIP8** has two classes of heterogeneous binding sites characterized by two K_d_ and two B_max_: K_d_(1) = 1.17 mM^-1^ and B_max_(1) = 0.8 μmol/mg for the higher affinity binding sites, and K_d_(2) = 200 μM^-1^ and B_max_(2) = 2.05 μmol/mg for the lower affinity binding sites. 

**Figure 7:**
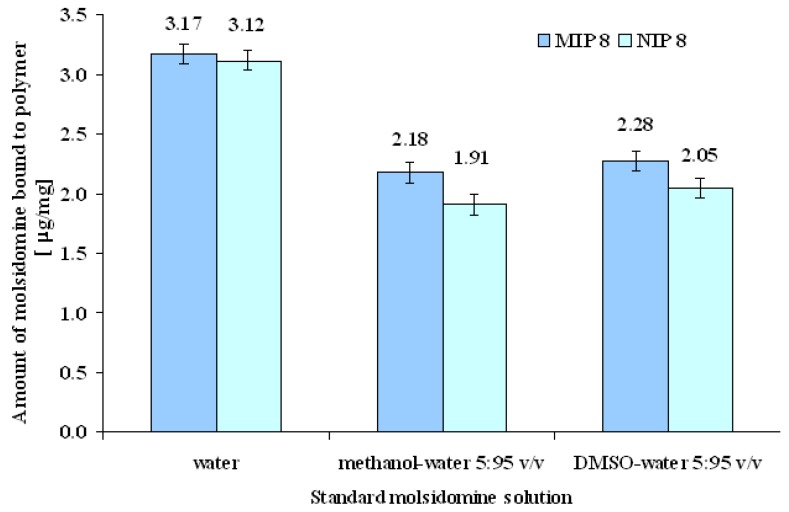
Amount of molsidomine bound per mg of polymer with different standard solutions applied for loading of molsidomine.

Additionally, evaluation of the capacity of **MIP8** and **NIP8** to recognize the template was performed in water at pH = 3.0 and pH = 7.0, in methanol-water and DMSO-water at molsidomine concentration of 0.07 μmol/mL. As stated above, the first binding experiments were performed in aqueous media (see Experimental, subsections c and e). The small difference between the quantity of molsidomine bound to **MIP8** and **NIP8** was explained by non-specific binding of molsidomine in water. Unfortunately, comparable small imprinting factors were obtained both in aqueous media at pH 3.0 as well as at pH 7.0. The tested range of pH values was limited by the stability of molsidomine [10]. Next, the binding of **MIP8** was tested by performing the same experiments in methanol-water (5:95 v/v) and in DMSO-water (5:95 v/v). The results are shown in Figure 7.

In methanol-water the amounts of bound molsidomine were 2.18 and 1.91 μg/mg for **MIP8** and **NIP8**, respectively. The imprinting factor was 1.14. Similar parameters of adsorption were observed in DMSO-water. The binding becomes higher than that observed in water. The possible explanation is that the organic solvents partially destroyed the non-specific interactions, which resulted in enhancement of the specific binding properties.

**Figure 8:**
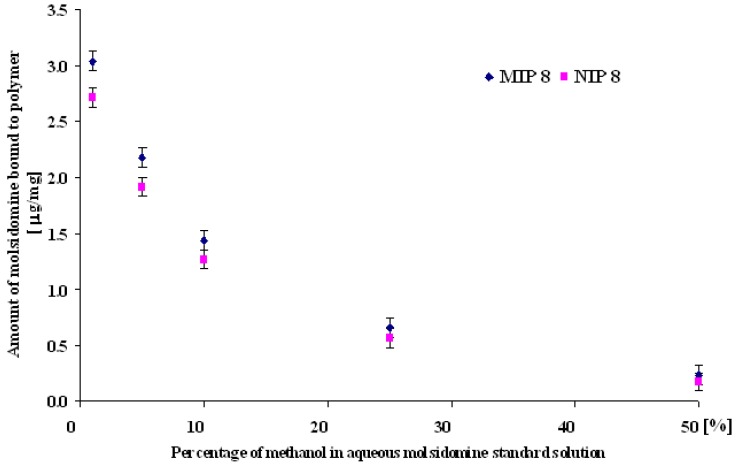
Amount of molsidomine bound to **MIP8** and **NIP8** in methanol-water solutions.

In order to evaluate the effect of methanol, the 0.07 μmol/mL molsidomine solution was prepared in methanol-water (1:99, 5:95, 10:90, 25:75, 50:50 v/v) and dynamic binding experiments were made (see Experimental, subsection f). The results are shown in Figure 8.

As can be seen, the small percentage of methanol (1-5 %) in aqueous molsidomine standard solution facilitates the distinction between the imprinted and the control polymer. This effect disappeared with the increasing fraction of methanol.

### MIP and NIP release properties

**Figure 9:**
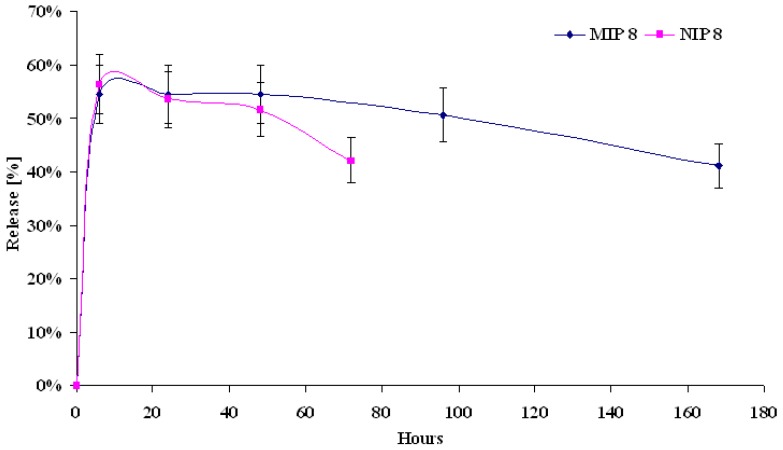
Release profiles of molsidomine from **MIP8** and **NIP8** at exchange of half of volume of releasing medium.

**MIP8** matrix, which is the most effective in template recognition was tested as a device for molsidomine delivery. Release studies were carried out in two parallel experiments for 25 mg of **MIP8** and **NIP8**. Both polymers were saturated with molsidomine after dynamic binding experiments (see Experimental, subsections g and h). The amounts of molsidomine bound to **MIP8** were higher than to **NIP8**, and were 146 and 128 μg, respectively. The release of molsidomine from **NIP8** was evaluated for 72 h and from **MIP8** it was extended to 168 h. The first measurements were made at an exchange of half of volume of the releasing medium. The results are shown in Figure 9.

We observed a difference between both profiles. After 6 h, **NIP8** released more molsidomine than **MIP8**, in spite of the lower initial amount of molsidomine. After 24 h **NIP8** released less molsidomine than **MIP8**, and the imprinted polymer released a constant amount of molsidomine for the next 72 h. After 72 h, **NIP8** released significantly less molsidomine (42 %) than **MIP8** (54 %). The results revealed the ability of **MIP8** to sustained releasing of molsidomine, even after one week.

**Figure 10:**
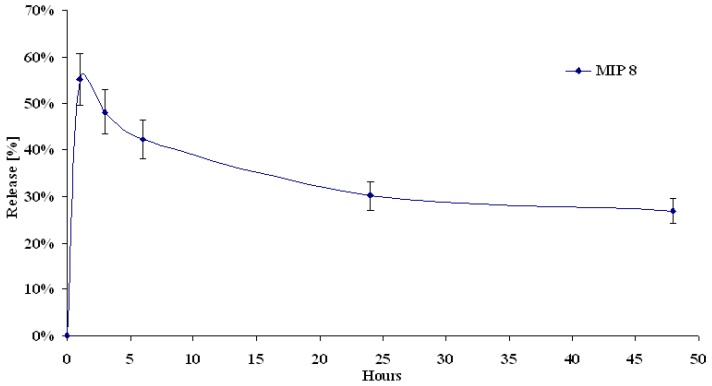
Release profile of molsidomine from **MIP8** at total volume exchange of releasing medium.

Next, we investigated the release characteristic of **MIP8** when the total volume of the releasing medium was exchanged. The release profile was presented as percentage of the initial amount of molsidomine (Figure 10).

The results showed that the imprinted polymer was still able to release a significant and nearly constant amount of molsidomine. Decrease of percentage of molsidomine released as a function of time, compared to results presented at Figure 9, is related with higher volume exchange of releasing medium.

## Conclusions

In this report we evaluated MIPs and NIPs obtained with eight different functional monomers. On the basis of the calculated imprinting factors, we chose **MIP8** and **NIP8** prepared on the basis of *N*,*N’*-diallyltartaramide for further investigations. Careful investigations of interactions in the prepolymeryzation complex were carried out. UV-VIS absorption spectra were recorded with a different molar ratio of molsidomine and *N*,*N’*-diallyltartaramide and Job’s plot was constructed. The most stable complex was formed with the molar ratio of template to functional monomer equal to 1:4. Binding characteristics of polymers were estimated with the Schatchard plot. The results showed heterogeneous binding sites with K_d_(1) = 1.17 mM^-1^ and B_max_(1) = 0.8 μmol/mg for the higher affinity binding sites, and K_d_(2) = 200 μM^-1^ and B_max_(2) = 2.05 μmol/mg for the lower affinity binding sites. We observed that the pH of loading solution had no influence on binding parameters. The addition of organic solvent such as methanol or DMSO improved the binding of molsidomine. The best result was obtained for the methanol-water solution (5:95 v/v). However, in organic medium the capacity of polymer matrix was significantly lower. Finally, we investigated release properties of **MIP8** and **NIP8**. We observed a difference between release profiles of MIP and NIP. The results revealed the ability of sustained release of molsidomine from the imprinted polymer even for one week. 

## Experimental

### General

Molsidomine and the following functional monomers: itaconic acid, methacrylamide, *N*,*N’*-methylenebisacrylamide, ethyl acrylate and 2-hydroxyethyl methacrylate, and *N*,*N’*-diallyltartaramide were obtained from Sigma-Aldrich (Germany). Acrylamide, methacrylic acid and ethylene glycol dimethacrylate were from Fluka (Germany). The polymerization reaction initiator 1,1’-azobiscyclo-hexanecarbonitrile, was provided by Fluka (Germany). Citric acid monohydrate and disodium hydrogen orthophosphate were from POCh (Poland). DMSO was obtained from Park Scientific Ltd. (United Kingdom). Methanol and acetone were from POCh (Poland). The monomers were purified prior to use by standard procedures (vacuum distilled or recrystallized from an appropriate solvent). All other reagents were used without further purification. Ultra-pure water was delivered from a Milli-Q purification system (Millipore, France). Dynamic binding experiments were perfomed in home-made solid-phase extraction vacuum manifold. Polypropylene 1 mL SPE columns with glass-fiber frits were used (Chromabond, Germany). UV-VIS measurements were performed with a UV-1605PC spectrophotometer (Shimadzu, Germany) at wave length of λ_max_ = 311 nm. The calibration curve of molsidomine as a function of absorbance (y) versus concentration (x) was performed in the range 0.01 - 0.15 μmol/mL. The calibration equation was y = 14.775 x + 0.005, r^2^ = 0.994, the limit of quantification (LOQ) was 2.30 μg/mL and the limit of detection (LOD) was 0.74 μg/mL.

### Stock solution of molsidomine

An accurately weighed amount of molsidomine (0.0242 g) was dissolved in methanol (10 mL). The stock solution of molsidomine (10 μmol/mL) was stored in dark at -20^o^C. The molsidomine standard solutions, prepared prior to use, were diluted with ultra-pure water or ultra-pure water with the appropriate amount of organic phase (methanol or DMSO) to give final concentrations of molsidomine.

### Preparation of molecularly imprinted and control polymers

The MIPs and NIPs were prepared by bulk polymerization. Briefly, molsidomine as a template (0.048 g, 0.2 mmol), selected functional monomer: methacrylic acid (0.069 g, 0.8 mmol), itaconic acid (0.104 g, 0.8 mmol), 2-hydroxyethyl methacrylate (0.104 g, 0.8 mmol), ethyl acrylate (0.080 g, 0.8 mmol), acrylamide (0.057 g, 0.8 mmol), methacrylamide (0.068 g, 0.8 mmol), *N*,*N’*-methylene-bisacrylamide (0.123 g, 0.8 mmol), *N*,*N’*-diallyltartaramide (0.183 g, 0.8 mmol), ethylene glycol dimethacrylate (0.634 g, 3.2 mmol) and 1,1’-azobiscyclohexanecarbonitrile (0.012 g, 1.2% mol) were dissolved in DMSO (0.671 mL) in a thick-walled glass tube. The appropriate homogeneous solutions were purged with nitrogen for ca. 3-5 min. Then the mixtures were incubated under a nitrogen atmosphere at 88^o^C for 24 h. The final bulk rigid polymers were ground in a mortar with a pestle and wet-sieved into particles below 100 μm diameter. Fine particles were removed by repeated precipitation in acetone. Finally, the particles were extracted to remove the molsidomine in the continuous extraction process in a Soxhlet apparatus (24-36 h, 80 mL methanol), and dried under vacuum at room temperature. The amount of molsidomine extracted from polymers was checked by UV-VIS measurements. The non-imprinted polymers were prepared and treated in the same way as the corresponding imprinted polymers, except the addition of molsidomine.

### Evaluation of polymers

*Stationary binding experiments*. The stationary binding experiments were performed in Eppendorf vials filled with 25 mg of dried-packed **MIP1**-**MIP8** and **NIP1**-**NIP8** particles. Next, to each vial was added:

(*a*) 1 mL of aqueous molsidomine standard solution of 0.07 or 0.7 μmol/mL;

(*b*) 1 mL of aqueous molsidomine standard solution of 0.01, 0.07, 0.15, 0.7, 3 and 6 μmol/mL;

(*c*) 1 mL of aqueous molsidomine standard solution of 0.07 μmol/mL, adjusted to pH 3.0 or pH 7.0 with phosphate-citrate buffer.

Next the vials were sealed and oscillated by a shaker (Heidolph, Germany) at room temperature for 6 h. Then the mixtures were centrifuged for 10 min. and an aliquot of solvent (0.1 mL) was used to detect the amount of molsidomine not bound to polymer by UV-VIS measurements. The amount of molsidomine bound to the particles was calculated by subtracting the unbound amount of molsidomine from the starting amount.

*Dynamic binding experiments*. The dynamic binding experiments were performed in 1 mL polypropylene SPE columns secured by glass-fiber frits and filled with 25 mg of dried-packed **MIP1**-**MIP8** and **NIP1**-**NIP8** particles. Then the particles were treated according to the following procedures:

(*d*) prewashed with methanol (1 mL), conditioned with buffered water (pH 5.0, phosphate-citrate buffer, 1 mL), loaded with successive portions of 1 mL of aqueous molsidomine standard solution of 0.07 μmol/mL or 0.7 μmol/mL until complete saturation of polymer;

(*e*) prewashed with methanol (1 mL), conditioned with methanol-water (5:95 v/v) or DMSO-water (5:95 v/v), 1 mL, loaded with successive portions of 1 mL of methanol-water (5:95 v/v) molsidomine standard solution of 0.07 μmol/mL or DMSO-water (5:95 v/v) molsidomine standard solution of 0.07 μmol/mL until complete saturation of polymer;

(*f*) prewashed with methanol (1 mL), conditioned with methanol-water (in the same proportion as loaded solutions, 1 mL), loaded one by one (1 mL) of methanol-water (1:99, 5:95, 10:90, 25:75, 50:50 v/v) molsidomine standard solution (0.07 μmol/mL) until complete saturation of polymer.

An aliquot of supernatant (0.1 mL or 0.4 mL) was used to analyze the amount of molsidomine unbound to polymer by UV-VIS spectroscopic measurements. The amount of molsidomine bound to the particles was calculated by subtracting the unbound amount from the starting amount of molsidomine in standard solution. 

All experiments were performed in triplicate.

Columns of **MIP8** and **NIP8** were also loaded with 0.7 μmol/mL molsidomine standard solution during dynamic binding protocol described above, then dried under vacuum and stored in dark (for no more than 48 h) for the release experiments.

*Release experiments*. The dried **MIP8** and **NIP8** particles loaded with molsidomine in dynamic binding experiments (described above) were quantitatively transferred to 10 mL polypropylene vials containing magnetic bar. The particles were dispersed with ultra-pure water (8 mL). Next the vials were sealed and the solutions were stirred for 48 h or 168 h. Then the samples of the solutions for determination by UV-VIS measurements of the amounts of molsidomine released were taken:

(*g*) 0.5 mL after 6, 24, 48, 72, 96 and 168 h;

(*h*) 2 mL after 1, 3, 6, 24 and 48 h.

The samples were replaced with the same volume of fresh ultra-pure water.

## References

[1] Komiyama M., Takeuchi T., Mukawa T., Asanuma H. (2003). Molecular Imprinting: From Fundamentals to Applications.

[2] Qiao F.X., Sun H.W., Yan H.Y., Row K.H. (2006). Molecularly imprinted polymers for solid-phase extraction. Chromatographia.

[3] Haupt K. (2003). Imprinted polymers – tailor-made mimics of antibodies and receptors. Chem. Commun..

[4] Alexander C., Davidson L., Hayes W. (2003). Imprinted polymers: artificial molecular recognition materials with applications in synthesis and catalysis. Tetrahedron.

[5] Alvarez-Lorenzo C., Concheiro A. (2004). Molecularly imprinted polymers for drug delivery. J. Chromatogr. B.

[6] Sellergren B., Allender C.J. (2005). Molecularly imprinted polymers: A bridge to advanced drug delivery. Adv. Drug Deliv. Rev..

[7] Puoci F., Iemma F., Cirillo G., Picci N., Matricardi P., Alhaique F. (2007). Molecularly imprinted polymers for 5-fluorouracil release in biological fluids. Molecules.

[8] Bayer C.L., Peppas N.A. (2008). Advances in recognitive, conductive and responsive delivery systems. J. Control. Release.

[9] Jantarat C., Tangthong N., Songkro S., Martin G.P., Suedee R. (2008). *S*-Propranolol imprinted polymer nanoparticle-on-microsphere composite porous cellulose membrane for the enantioselectively controlled delivery of racemic propanolol. Int. J. Pharm..

[10] Streel B., Ceccato A., Peerboom C., Zimmer C., Sibenaler R., Maes P. (1998). Determination of molsidomine and its active metabolite in human plasma using liquid chromatography with tandem mass spectric detection. J. Chromatogr. A.

[11] Schönafinger K. (1999). Heterocyclic NO prodrugs. Il Farmaco.

[12] Frisch M.J., Trucks G.W., Schlegel H.B., Scuseria G.E., Robb M.A., Cheeseman J.R., Montgomery J.A., Vreven T., Kudin K.N., Burant J.C., Millam J.M., Iyengar S.S., Tomasi J., Barone V., Mennucci B., Cossi M., Scalmani G., Rega N., Petersson G.A., Nakatsuji H., Hada M., Ehara M., Toyota K., Fukuda R., Hasegawa J., Ishida M., Nakajima T., Honda Y., Kitao O., Nakai H., Klene M., Li X., Knox J.E., Hratchian H.P., Cross J.B., Adamo C., Jaramillo J., Gomperts R., Stratmann R.E., Yazyev O., Austin A.J., Cammi R., Pomelli C., Ochterski J.W., Ayala P.Y., Morokuma K., Voth G.A., Salvador P., Dannenberg J.J., Zakrzewski V.G., Dapprich S., Daniels A.D., Strain M.C., Farkas O., Malick D.K., Rabuck A.D., Raghavachari K., Foresman J.B., Ortiz J.V., Cui Q., Baboul A.G., Clifford S., Cioslowski J., Stefanov B.B., Liu G., Liashenko A., Piskorz P., Komaromi I., Martin R.L., Fox D.J., Keith T., Al-Laham M.A., Peng C.Y., Nanayakkara A., Challacombe M., Gill P.M.W., Johnson B., Chen W., Wong M.W., Gonzalez C., Pople J.A. (2003). Gaussian 03, Revision B.02.

[13] Improta R., Santoro F., Barbier C., Giordano F., Del Re G. (1998). On the geometry of 3-amino-sydnones. J. Mol. Struct. (THEOCHEM).

[14] Moss G.P., Smith P.A.S., Tavernier D. (1995). Glossary of class names of organic compounds and reactive intermediates based on structure. Pure Appl. Chem..

[15] Laudy D., Tetrat F., Truant E., Blach P., Fourmentin S., Surpateanu G. (2007). Development of a competitive continuous variation plot for the determination of inclusion compounds stoichiometry. J. Incl. Phenom. Macrocycl. Chem..

[16] Striegler S., Dittel M. (2003). Evaluation of new strategies to prepare templated polymers with sufficient oligosaccharide recognition capacity. Anal. Chim. Acta.

[17] Svenson J., Karlsson J.G., Nicholls I.A. (2004). ^1^H Nuclear magnetic resonance study of the molecular imprinting of (-)nicotine: template self-association, a molecular basis for cooperative ligand binding. J. Chrom. A.

[18] Breton F., Delepee R., Jegourel D., Deville-Bonne D., Agrofoglio L.A. (2008). Selective adenosine-5’-monophosphate uptake by water-compatible molecularly imprinted polymer. Anal. Chim. Acta.

[19] Sun Z., Schüssler W., Sengl M., Niessner R., Knopp D. (2008). Selective trace analysis of diclofenac in surface and wastewater samples using solid-phase extraction with a new molecularly imprinted polymer. Anal. Chim. Acta.

